# Association between the stress hyperglycemia ratio and 28-day all-cause mortality in critically ill patients with sepsis: a retrospective cohort study and predictive model establishment based on machine learning

**DOI:** 10.1186/s12933-024-02265-4

**Published:** 2024-05-09

**Authors:** Fengjuan Yan, Xiehui Chen, Xiaoqing Quan, Lili Wang, Xinyi Wei, Jialiang Zhu

**Affiliations:** 1 Department of Geriatrics, Shenzhen Longhua District Central Hospital, Shenzhen, Guangdong China; 2 Department of Cardiology, Affiliated Hospital of Shandong University of Traditional Chinese Medicine, Jinan, Shandong China; 3 Department of Cardiology, The Third Hospital of Jinan, Jinan, Shandong China; 4 The First Affiliated Hospital of Jinan University, Guangzhou, Guangdong China

**Keywords:** Sepsis, Stress hyperglycemia ratio, Critical illness, Boruta algorithm, Machine learning

## Abstract

**Background:**

Sepsis is a severe form of systemic inflammatory response syndrome that is caused by infection. Sepsis is characterized by a marked state of stress, which manifests as nonspecific physiological and metabolic changes in response to the disease. Previous studies have indicated that the stress hyperglycemia ratio (SHR) can serve as a reliable predictor of adverse outcomes in various cardiovascular and cerebrovascular diseases. However, there is limited research on the relationship between the SHR and adverse outcomes in patients with infectious diseases, particularly in critically ill patients with sepsis. Therefore, this study aimed to explore the association between the SHR and adverse outcomes in critically ill patients with sepsis.

**Methods:**

Clinical data from 2312 critically ill patients with sepsis were extracted from the MIMIC-IV (2.2) database. Based on the quartiles of the SHR, the study population was divided into four groups. The primary outcome was 28-day all-cause mortality, and the secondary outcome was in-hospital mortality. The relationship between the SHR and adverse outcomes was explored using restricted cubic splines, Cox proportional hazard regression, and Kaplan‒Meier curves. The predictive ability of the SHR was assessed using the Boruta algorithm, and a prediction model was established using machine learning algorithms.

**Results:**

Data from 2312 patients who were diagnosed with sepsis were analyzed. Restricted cubic splines demonstrated a "U-shaped" association between the SHR and survival rate, indicating that an increase in the SHR is related to an increased risk of adverse events. A higher SHR was significantly associated with an increased risk of 28-day mortality and in-hospital mortality in patients with sepsis (HR > 1, P < 0.05) compared to a lower SHR. Boruta feature selection showed that SHR had a higher Z score, and the model built using the rsf algorithm showed the best performance (AUC = 0.8322).

**Conclusion:**

The SHR exhibited a U-shaped relationship with 28-day all-cause mortality and in-hospital mortality in critically ill patients with sepsis. A high SHR is significantly correlated with an increased risk of adverse events, thus indicating that is a potential predictor of adverse outcomes in patients with sepsis.

**Supplementary Information:**

The online version contains supplementary material available at 10.1186/s12933-024-02265-4.

## Introduction

Infectious diseases have long been at the forefront of global health challenges. Sepsis, which is a severe complication of infections, is closely associated with abnormal immune responses. Sepsis often leads to multiorgan dysfunction, thereby posing a threat to the lives of affected individuals [1]. Despite significant progress in the management of infectious diseases due to widespread antibiotic use and advancements in medical technology, the incidence of sepsis remains high, and its mortality rate is a cause for concern [2].

Stress hyperglycemia is a physiological phenomenon characterized by a significant increase in blood glucose levels in response to physiological or pathological stress. This phenomenon is often associated with the regulation of hormone secretion, immune responses, and neural system activity. Stress hyperglycemia is typically linked to the release of hormones such as adrenaline and cortisol; furthermore, it is associated with insulin resistance and increased hepatic gluconeogenesis [3]. In some cases, stress hyperglycemia is an adaptive physiological response that helps to provide energy for the body to cope with physiological or pathological stress. However, for patients with chronic diseases or those facing prolonged stress, this hyperglycemic state may be associated with unfavorable clinical outcomes. A previous study indicated an independent correlation between acute hyperglycemia upon admission and adverse outcomes in the early and late stages of acute myocardial infarction (AMI) [4]. To explain and evaluate the impact of background blood glucose, researchers introduced HbA1c as a baseline glucose level when assessing stress hyperglycemia, and they proposed the stress hyperglycemia ratio (SHR) as a new indicator to assess a more accurate representation of acute hyperglycemia [5]. Previous studies have shown that in patients with acute coronary syndrome, critically ill patients, and patients with critical myocardial infarction, a higher SHR is associated with an increased risk of adverse outcomes [6–8].

Due to the abnormal activation of the immune system and the massive release of various cytokines in patients with sepsis, they often experience a state of stress [9]. However, it is currently unclear whether the SHR is correlated with adverse outcomes in critically ill patients with sepsis. Therefore, this study aimed to assess the association between the SHR and adverse outcomes in critically ill patients with sepsis.

## Methods

### Data source

The data utilized in this study are derived from MIMIC-IV2.2, which is an electronic health record dataset encompassing over 50,000 patients admitted to the intensive care unit (ICU) at Beth Israel Deaconess Medical Center (BIDMC) in Boston, Massachusetts, from 2008 to 2019. The Institutional Review Board of BIDMC granted a waiver of informed consent and approved the sharing of research resources. The author (JLZ) obtained access to the database (certificate number: 45848364).

### Inclusion and exclusion criteria

Inclusion Criteria:

1. Patients aged between 18 and 90 years.
2. Patients diagnosed with sepsis according to the recommendations of the Third International Consensus Definitions for Sepsis and Septic Shock (Sepsis-3).

Exclusion Criteria:

1. Patients with an ICU stay of less than 24 h.
2. Missing serum glucose and glycated hemoglobin in the first laboratory test.
3. For patients with multiple ICU admissions, only data from the first hospitalization were included.

### Outcome

The primary outcome was 28-day all-cause mortality, and the secondary outcome was in-hospital mortality.

### Data extraction

Data extraction was performed using pgAdmin software. Patient characteristics, including age, sex, and weight, were collected. Information on comorbidities, such as hypertension, type 2 diabetes, type 1 diabetes, heart failure, malignancy, chronic kidney disease (CKD), stroke, pneumonia and septic shock, was extracted based on the International Classification of Diseases coding system. Vital signs (heart rate, systolic blood pressure (SBP), respiratory rate, arterial oxygen saturation (SaO2) and laboratory tests [red blood cell count (RBC), white blood cell count (WBC), platelet count (PLT), serum sodium, serum potassium, serum calcium, anion gap, pH, carbon dioxide pressure (PaCO2), arterial oxygen pressure (PaO2), lactate, prothrombin time international normalized ratio (INR), total bilirubin, aspartate aminotransferase (AST), blood urea nitrogen, serum creatinine, serum glucose, and glycosylated hemoglobin (HbA1c)] were extracted. The use of steroids (glucocorticoids) was also extracted. Additionally, the severity of illness was assessed using the sequential organ failure assessment (SOFA). The stress hyperglycemia ratio (SHR) is defined as the index calculated using the following formula: SHR = (admission blood glucose (mg/dl))/(28.7 × HbA1c(%) − 46.7)] [5].

### Statistical analysis

As the current study is a retrospective analysis, no sample size calculations were conducted. Variables with a missing data rate exceeding 20% were excluded, and for those with less than a missing data rate below 20%, multiple imputation was employed. The variance inflation factor (VIF) was used to assess multicollinearity among variables. Variables with a VIF exceeding 5 were removed due to multicollinearity concerns. Patients were categorized into four groups based on quartiles of the SHR. Normally distributed continuous variables are presented as the means (standard deviations [SDs]) and were analyzed using analysis of variance (ANOVA). Nonnormally distributed variables were analyzed using the Mann‒Whitney U test or the Kruskal‒Wallis test. Categorical variables are expressed as numbers and percentages and were analyzed using the χ^2^ test or Fisher’s exact test. Kaplan‒Meier survival curves were used to compare the 28-day survival rates among the four groups according to the log-rank test. Proportional hazard regression models (Cox regression models) were used to assess the hazard ratio (HR) and 95% confidence interval (95% CI) for event occurrence. Model I was unadjusted for covariates. To adjust for the impacts of patient general condition and vital signs, SOFA scores, and steroid use on outcomes, Model II was adjusted for age, weight, sex, heart rate, respiratory rate, systolic blood pressure, SOFA scores, and steroid use (glucocorticoids). The time-dependent receiver operating characteristic (ROC) curve was used to compare the SHR and laboratory indicators of each continuous variable. A two-tailed P value < 0.05 was considered to indicate statistical significance. Statistical analyses were conducted using R software (version 4.3.1).

### Restricted cubic splines

In this study, we collected data on survival (the outcome variable); the SHR (the continuous predictor variable); and age, weight, heart rate, SBP, respiratory rate, sex, SOFA scores and steroid use (the covariates). The potential nonlinear relationships between the change in the SHR and survival were examined by a Cox regression model with restricted cubic spline (RCS). Knots between 3 and 7 were tested, and the model with the lowest Akaike information criterion value was selected for the RCS.

### Subgroup analysis

A subgroup analysis was conducted based on prespecified criteria, including age, sex, and type 2 diabetes status, and univariate analysis and multivariate analysis were performed. The multivariate analysis was adjusted for age, weight, sex, heart rate, respiratory rate, systolic blood pressure, SOFA scores, and steroids (glucocorticoids). The multivariable analysis of the male and female subgroups were not adjusted for sex. Patients were stratified into two groups based on age (< 65 years and ≥ 65 years). Cox proportional hazards regression analysis was performed for each subgroup, and the results were visually presented using forest plots, illustrating hazard ratios (HRs) and 95% confidence intervals (CIs).

### Establishment and validation of the prediction models

Boruta’s algorithm is a method used to determine the most important features in a dataset. It identifies importance by comparing the Z value of each feature with the Z value of the corresponding “shadow feature”. In the algorithm, all real features are copied and shuffled, and then the Z value of each feature is obtained through the random forest model. Additionally, the Z values of the ‘shadow features’ are generated by randomly shuffling the real features [10]. If the Z value of a true feature is significantly higher than the maximum Z value of the shadow feature in multiple independent tests, the true feature is marked as “important” (green area), also known as an acceptable variable. Otherwise, it is marked as “unimportant” (red area), also known as unacceptable variables. Acceptable variables are variables that are retained during the feature selection process and are considered to contribute to the performance of the model. Unacceptable variables are excluded from the final feature selection by the algorithm because they fail to show predictive power for the target variable during the feature selection process. In addition, the Boruta algorithm was also used to explore the importance of the SHR as a predictor variable.

Acceptable variables are incorporated into the machine learning algorithm. The dataset was divided into training and validation sets at a 7:3 ratio. The filtered variables were individually analyzed using the Cox Proportional Hazards Survival Learner (coxph) algorithm, Rpart Survival Trees Survival Learner (dt) algorithm, Survival DeepSurv Learner (deepsurv) algorithm, Survival Random Forest SRC Learner (rsf), and Extreme Gradient Boosting Survival Learner (xgboost) algorithm to predict the 28-day mortality risk in critically ill sepsis patients. Hyperparameter tuning is performed during the establishment of machine learning models. The training and validation sets were utilized for model establishment and evaluation, respectively. The ROC curve and its corresponding area under the curve (AUC) were used to determine model performance. Decision curve analysis (DCA) was employed for assessing clinical effectiveness, while calibration curves were used to evaluate the accuracy of the model in predicting absolute risk.

## Results

### Baseline characteristics

**Fig. 1:**
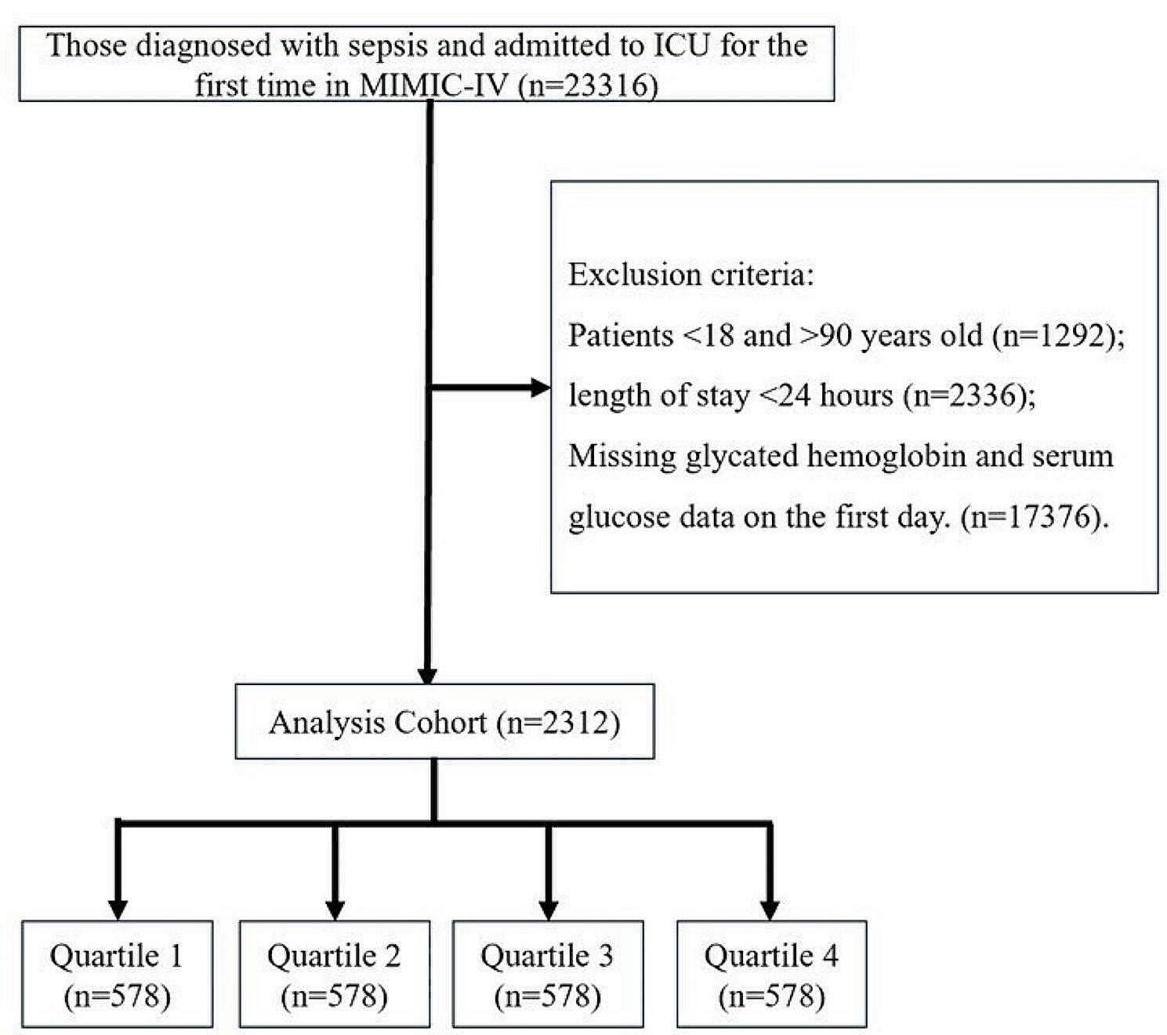
Selection of the study population from the MIMIC-IV database

Data from 2,312 patients diagnosed with sepsis were extracted from the MIMIC-IV (Fig. 1). Table S1 displays the variance inflation factors, indicating that there was no multicollinearity among the variables. Figure S1 illustrates the proportions of missing data for each variable, and Table 1 presents the baseline characteristics of the study subjects. There were 1396 males (60.38%), 1046 (45.24%) patients with hypertension, 906 (39.19%) patients with type 2 diabetes, 74 (3.2%) patients with type 1 diabetes, 805 (34.82%) patients with heart failure, 248 (10.73%) patients with malignancy, 450 (19.46%) patients with CKD, 327 (14.14%) patients with stroke, 372 (16.09%) patients with septic shock and 925 (40.01%) patients with pneumonia. Patients were divided into quartiles: quartile 1 (0.21 ≤ SHR < 0.915), quartile 2 (0.915 ≤ SHR < 1.14), quartile 3 (1.14 ≤ SHR < 1.45), and quartile 4 (1.45 ≤ SHR ≤ 7.41), with each group consisting of 578 individuals. Patients in Quartile 4 exhibited a higher heart rate, white blood cell count, serum potassium, anion gap, total bilirubin, aspartate aminotransferase, blood urea nitrogen, serum creatinine, lactate, SOFA score, and steroid use ratio as well as lower serum sodium, pH, and PaO_2_ levels.

**Table 1:** Patient demographics and baseline characteristics

Characteristic (Mean ± SD)	SHR					p value^2^
	Overall, N = 2312^1^	Quartile 1, N = 578^1^	Quartile 2, N = 578^1^	Quartile 3, N = 578^1^	Quartile 4, N = 578^1^	
Age (years)	65 ± 15	65 ± 14	65 ± 15	66 ± 15	64 ± 14	0.244
Weight (kg)	87 ± 26	87 ± 29	87 ± 24	86 ± 24	87 ± 25	0.588
SBP (mmHg)	127 ± 26	127 ± 24	127 ± 25	128 ± 26	125 ± 26	0.169
Heart Rate	90 ± 21	86 ± 20	88 ± 21	92 ± 20	94 ± 21	< 0.001
Respiratory rate	20.0 ± 6.2	19.4 ± 6.3	20.0 ± 6.0	20.3 ± 6.1	20.3 ± 6.4	0.058
WBC (10^9/L)	13 ± 7	11 ± 7	12 ± 7	14 ± 8	15 ± 8	< 0.001
RBC (10^9/L)	3.82 ± 0.79	3.86 ± 0.77	3.82 ± 0.74	3.85 ± 0.79	3.75 ± 0.86	0.078
PLT (10^9/L)	213 ± 103	219 ± 109	208 ± 95	214 ± 108	210 ± 101	0.241
Sodium (mmol/L)	138.4 ± 5.6	139.2 ± 4.9	138.5 ± 5.2	138.4 ± 5.5	137.8 ± 6.6	< 0.001
Potassium(mmol/L)	4.22 ± 0.81	4.19 ± 0.75	4.17 ± 0.78	4.17 ± 0.79	4.36 ± 0.90	< 0.001
Total calcium (mmol/L)	8.34 ± 0.86	8.39 ± 0.79	8.38 ± 0.79	8.35 ± 0.92	8.25 ± 0.92	0.030
Anion gap (mmol/L)	15.9 ± 4.4	14.9 ± 4.0	15.2 ± 3.9	16.0 ± 4.0	17.4 ± 5.1	< 0.001
PH	7.37 ± 0.10	7.38 ± 0.08	7.39 ± 0.09	7.37 ± 0.09	7.33 ± 0.11	< 0.001
PaCO2 (mmHg)	41 ± 11	42 ± 11	41 ± 10	41 ± 11	42 ± 11	0.066
PaO2 (mmHg)	149 ± 111	157 ± 112	161 ± 120	147 ± 107	132 ± 103	< 0.001
Lactate (mmol/L)		1.71 ± 1.38	1.77 ± 1.38	2.02 ± 1.46	2.92 ± 2.26	< 0.001
SaO2 (%)	96.86 ± 3.94	96.94 ± 3.61	96.96 ± 3.53	96.86 ± 3.45	96.70 ± 4.98	0.657
INR	1.40 ± 0.64	1.39 ± 0.59	1.39 ± 0.72	1.35 ± 0.54	1.46 ± 0.68	0.039
Total bilirubin	1.23 ± 3.30	0.83 ± 1.09	1.21 ± 3.26	1.28 ± 3.62	1.61 ± 4.28	< 0.001
AST (U/l)	190 ± 944	138 ± 573	155 ± 856	139 ± 567	326 ± 1,470	< 0.001
Urea nitrogen (mg/dL)	29 ± 22	27 ± 22	26 ± 20	28 ± 24	33 ± 23	< 0.001
Creatinine (mg/dL)	1.59 ± 1.68	1.49 ± 1.55	1.51 ± 1.59	1.47 ± 1.50	1.89 ± 2.00	< 0.001
SOFA	5.5 ± 3.3	4.8 ± 3.1	4.9 ± 3.2	5.5 ± 3.3	6.6 ± 3.5	< 0.001
Blood glucose (mg/dL)	177 ± 104	115 ± 45	139 ± 50	172 ± 62	283 ± 136	< 0.001
HbA1c (%)	6.60 ± 1.92	7.09 ± 2.30	6.37 ± 1.66	6.33 ± 1.70	6.62 ± 1.86	< 0.001
Gender						0.128
Female	916 (39.62%)	233 (40.31%)	209 (36.16%)	248 (42.91%)	226 (39.10%)	
Male	1396 (60.38%)	345 (59.69%)	369 (63.84%)	330 (57.09%)	352 (60.90%)	
Hypertension						0.004
No	1266 (54.76%)	314 (54.33%)	310 (53.63%)	291 (50.35%)	351 (60.73%)	
Yes	1046 (45.24%)	264 (45.67%)	268 (46.37%)	287 (49.65%)	227 (39.27%)	
Diabetes II						< 0.001
No	1406 (60.81%)	332 (57.44%)	392 (67.82%)	366 (63.32%)	316 (54.67%)	
Yes	906 (39.19%)	246 (42.56%)	186 (32.18%)	212 (36.68%)	262 (45.33%)	
Diabetes I						< 0.001
No	2240 (96.88%)	553 (95.67%)	570 (98.61%)	572 (98.96%)	545 (94.29%)	
Yes	72 (3.11%)	25 (4.32%)	8 (1.38%)	6 (1.04%)	33 (5.71%)	
Heart failure						0.146
No	1507 (65.18%)	369 (63.84%)	391 (67.65%)	388 (67.13%)	359 (62.11%)	
Yes	805 (34.82%)	209 (36.16%)	187 (32.35%)	190 (32.87%)	219 (37.89%)	
Malignant tumor						0.217
No	2064 (89.27%)	515 (89.10%)	515 (89.10%)	506 (87.54%)	528 (91.35%)	
Yes	248 (10.73%)	63 (10.90%)	63 (10.90%)	72 (12.46%)	50 (8.65%)	
CKD						0.091
No	1862 (80.54%)	468 (80.97%)	464 (80.28%)	482 (83.39%)	448 (77.51%)	
Yes	450 (19.46%)	110 (19.03%)	114 (19.72%)	96 (16.61%)	130 (22.49%)	
Stroke						0.001
No	1985 (85.86%)	506 (87.54%)	486 (84.08%)	475 (82.18%)	518 (89.62%)	
Yes	327 (14.14%)	72 (12.46%)	92 (15.92%)	103 (17.82%)	60 (10.38%)	
Pneumonia						0.014
No	1387 (59.99%)	335 (57.96%)	380 (65.74%)	338 (58.48%)	334 (57.79%)	
Yes	925 (40.01%)	243 (42.04%)	198 (34.26%)	240 (41.52%)	244 (42.21%)	
Septic shock						0.003
No	1940 (83.91%)	496 (85.81%)	498 (86.16%)	489 (84.60%)	457 (79.07%)	
Yes	372 (16.09%)	82 (14.19%)	80 (13.84%)	89 (15.40%)	121 (20.93%)	
Steroids (glucocorticoid)						0.029
No	1958 (84.68%)	502 (86.85%)	503 (87.02%)	476 (82.35%)	477 (82.53%)	
Yes	354 (15.31%)	76 (13.15%)	75 (12.98%)	102 (17.65%)	101 (17.47%)	

SHR: Quartile 1 (0.21–0.915), Quartile 2 (0.915–1.14), Quartile 3 (1.14–1.45), and Quartile 4 (1.45–7.41).

*SBP* systolic blood pressure, *WBC* white blood cell count, *RBC* red blood cell count, *PLT* platelet count, *PaCO2* carbon dioxide pressure, *PaO2* arterial oxygen pressure, *SaO2* arterial oxygen saturation, *INR* prothrombin time international normalized ratio, *AST* aspartate aminotransferase, *SOFA* sequential organ failure assessment, *HbA1c* glycosylated hemoglobin, *CKD* chronic kidney disease, *Diabetes I* type 1 diabetes, *Diabetes II* type 2 diabetes, *SHR* stress hyperglycemia ratio

### Clinical outcomes

**Fig. 2:**
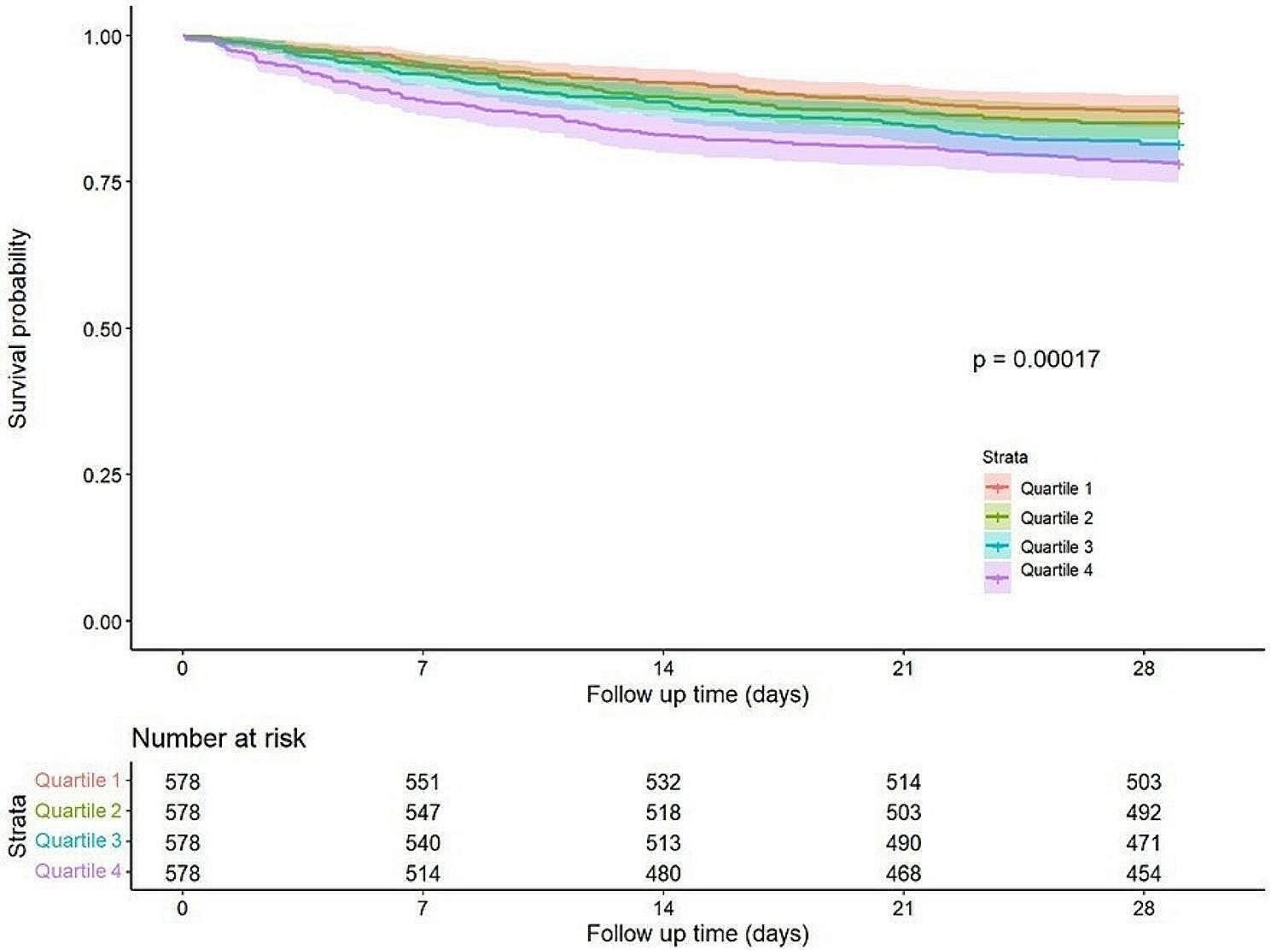
28-day KM survival curve. KM curves showing the survival rates at 28 days for each quartile. SHR: Quartile 1 (0.21–0.915), Quartile 2 (0.915–1.14), Quartile 3 (1.14–1.45), and Quartile 4 (1.45–7.41)

Regarding 28-day mortality and in-hospital mortality, Quartile 4 had higher mortality (Table 2). In the Cox regression analysis, the results of Models I and II showed that with Quartile 1 as the reference, the risk of mortality in Quartile 3 and Quartile 4 increased significantly (Table 3, Table 4). Kaplan–Meier curves revealed that patients in Quartile 4 had the lowest 28-day survival probability, and the difference was significant (Fig. 2).

**Table 2:** 28-day all-cause mortality and in-hospital mortality

SHR	Quartile 1	Quartile 2	Quartile 3	Quartile 4	P value
Mortality, n (%)					
28-day all-cause mortality	75 (13%)	86 (15%)	107 (19%)	126 (22%)	< 0.001
In-hospital mortality	67 (12%)	73 (13%)	86 (15%)	111 (19%)	0.001

SHR: Quartile 1 (0.21–0.915), Quartile 2 (0.915–1.14), Quartile 3 (1.14–1.45), and Quartile 4 (1.45–7.41)

**Table 3:** Cox regression model (28-day all-cause mortality)

SHR	Unadjusted HR (95% CI)	P value	Adjusted HR (95% CI)	P value
Quartile 1	Reference		Reference	
Quartile 2	1.16 (0.85–1.58)	0.340	1.14 (0.82–1.59)	0.430
Quartile 3	1.47 (1.09–1.97)	0.011	1.45 (1.08–1.95)	0.015
Quartile 4	1.80 (1.35–2.40)	< 0.001	1.84 (1.38–2.46)	< 0.001

SHR: Quartile 1 (0.21–0.915), Quartile 2 (0.915–1.14), Quartile 3 (1.14–1.45), and Quartile 4 (1.45–7.41)

**Table 4:** Cox regression model (in-hospital mortality)

SHR	Unadjusted HR (95% CI)	P value	Adjusted HR (95% CI)	P value
Quartile 1	Reference		Reference	
Quartile 2	1.17 (0.84–1.64)	0.350	1.09 (0.76–1.56)	0.630
Quartile 3	1.52 (1.11–2.11)	0.010	1.29 (1.02–2.01)	0.031
Quartile 4	1.87 (1.37–2.56)	< 0.001	1.84 (1.32–2.58)	< 0.001

SHR: Quartile 1 (0.21–0.915), Quartile 2 (0.915–1.14), Quartile 3 (1.14–1.45), and Quartile 4 (1.45–7.41)

### Restricted cubic spline

**Fig. 3:**
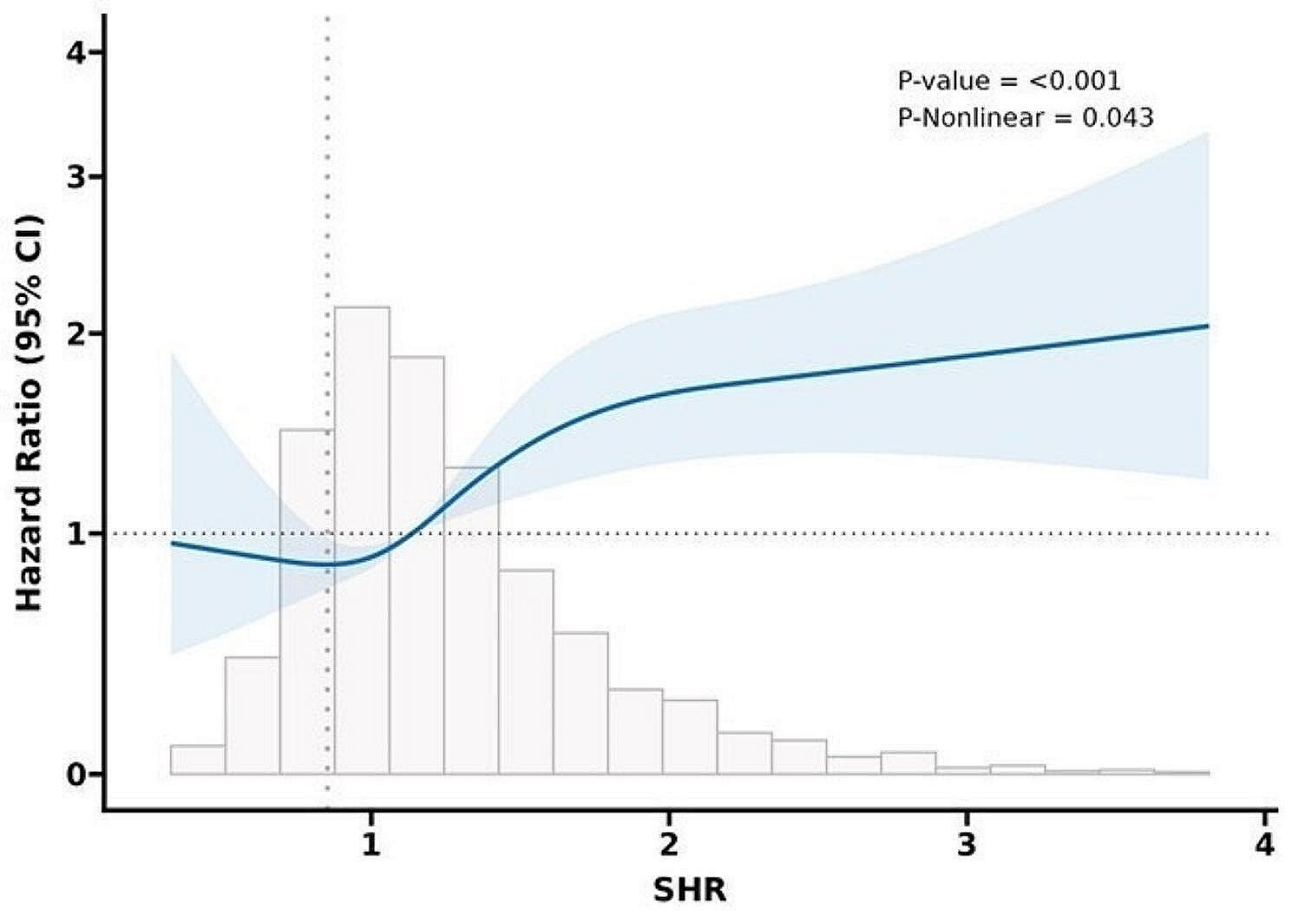
RCS analysis of 28-day all-cause mortality. Curves represent estimated adjusted hazard ratios, and shaded ribbons represent 95% confidence intervals. The vertical dotted line represents the lowest point of the curve (SHR = 0.85), which represents the lowest hazard ratio. The horizontal dashed line represents a hazard ratio of 1.0. *HR* hazard ratio, *CI* confidence interval

**Fig. 4:**
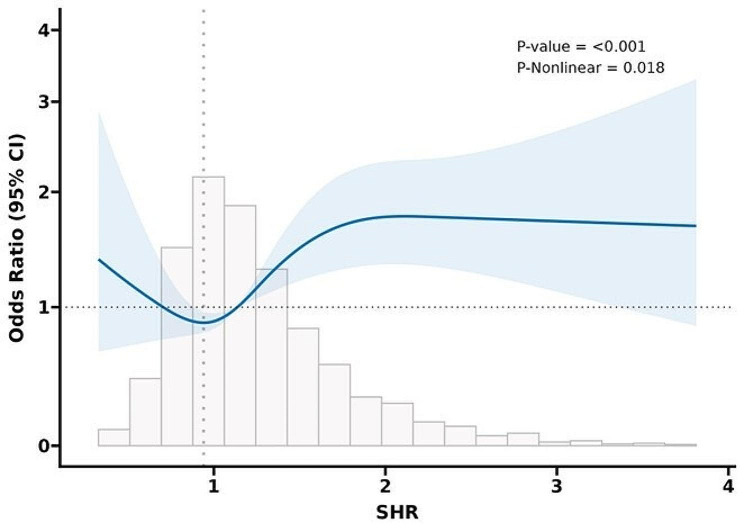
RCS results for in-hospital mortality. Curves represent estimated adjusted hazard ratios, and shaded ribbons represent 95% confidence intervals. The vertical dotted line represents the lowest point of the curve, which represents the lowest hazard ratio. The horizontal dashed line represents a hazard ratio of 1.0. *HR* hazard ratio; CI, confidence interval

The RCS analysis was adjusted for the effects of age, weight, heart rate, systolic blood pressure, respiratory rate, sex, SOFA scores and steroid use. The RCS analysis for 28-day all-cause mortality (Fig. 3) and in-hospital mortality (Fig. 4) both indicated a U-shaped association between the SHR and mortality risk. The turning point of the RCS curve is approximately SHR = 0.85, representing the inflection point in the relationship between the SHR and mortality risk. According to the Cox proportional hazards regression model, the turning point lies in Quartile 1, where the mortality risk is minimized, thus demonstrating consistency between the two results.

### Subgroup analysis

**Fig. 5:**
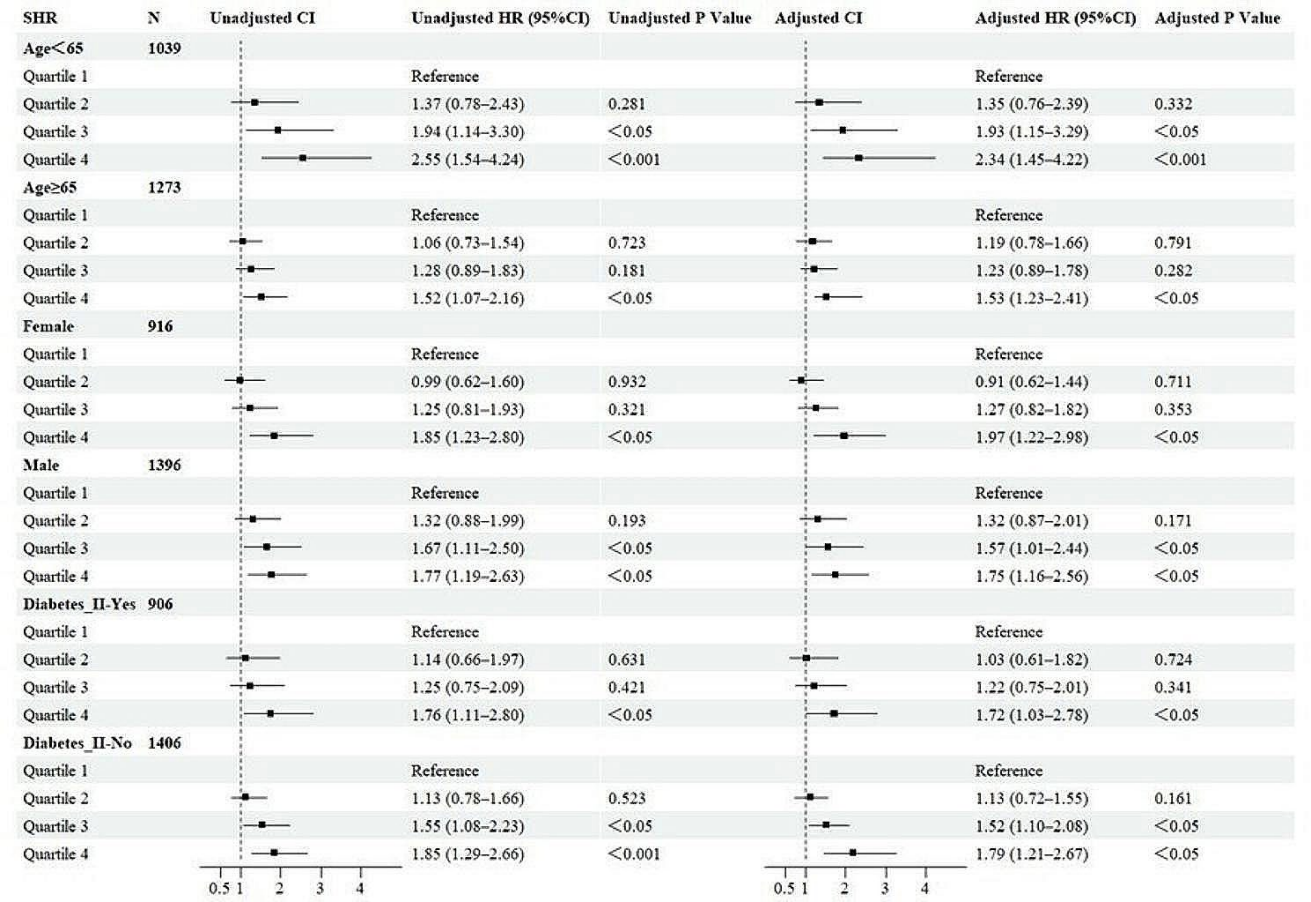
Subgroup forest plot for 28-day all-cause mortality. Adjusted for age, weight, sex, heart rate, respiratory rate, systolic blood pressure, SOFA scores, and use of steroids (glucocorticoids). SHR: Quartile 1 (0.21–0.915), Quartile 2 (0.915–1.14), Quartile 3 (1.14–1.45), and Quartile 4 (1.45–7.41). *Diabetes I* type 1 diabetes, *Diabetes II* type 2 diabetes, *HR* hazard ratio, *CI* confidence interval

The results presented a subgroup analysis of 28-day all-cause mortality (Fig. 5). In subgroups defined by age < 65, age ≥ 65, male sex, female sex, and type 2 diabetes status, Quartile 4 consistently demonstrated a greater risk of mortality, regardless of whether covariates were adjusted. This finding indicates that, irrespective of baseline levels, a higher SHR is associated with an increased 28-day mortality risk in critically ill sepsis patients (HR > 1 in each subgroup, P < 0.05).

### Boruta Algorithm

**Fig. 6:**
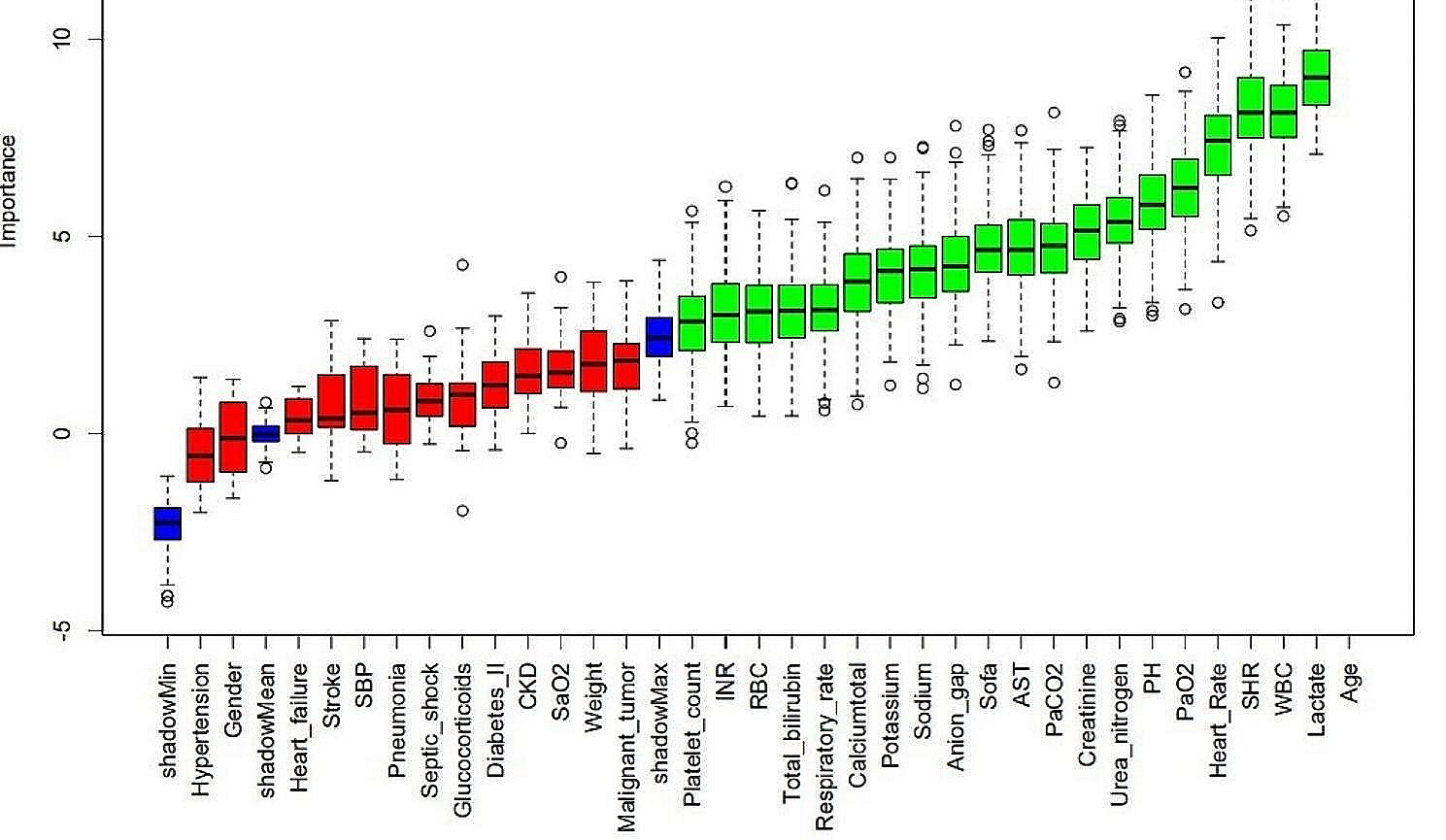
Feature selection based on the Boruta algorithm. The horizontal axis is the name of each variable, and the vertical axis is the Z value of each variable. The box plot shows the Z value of each variable during model calculation. The green boxes represent important variables, and the red boxes represent unimportant variables

Figure 6 shows the feature selection results based on the Boruta algorithm. Variables in the green area are identified as important features, and variables in the red area are unimportant features in the Boruta algorithm.

### Establishment and validation of the prediction model

**Fig. 7:**
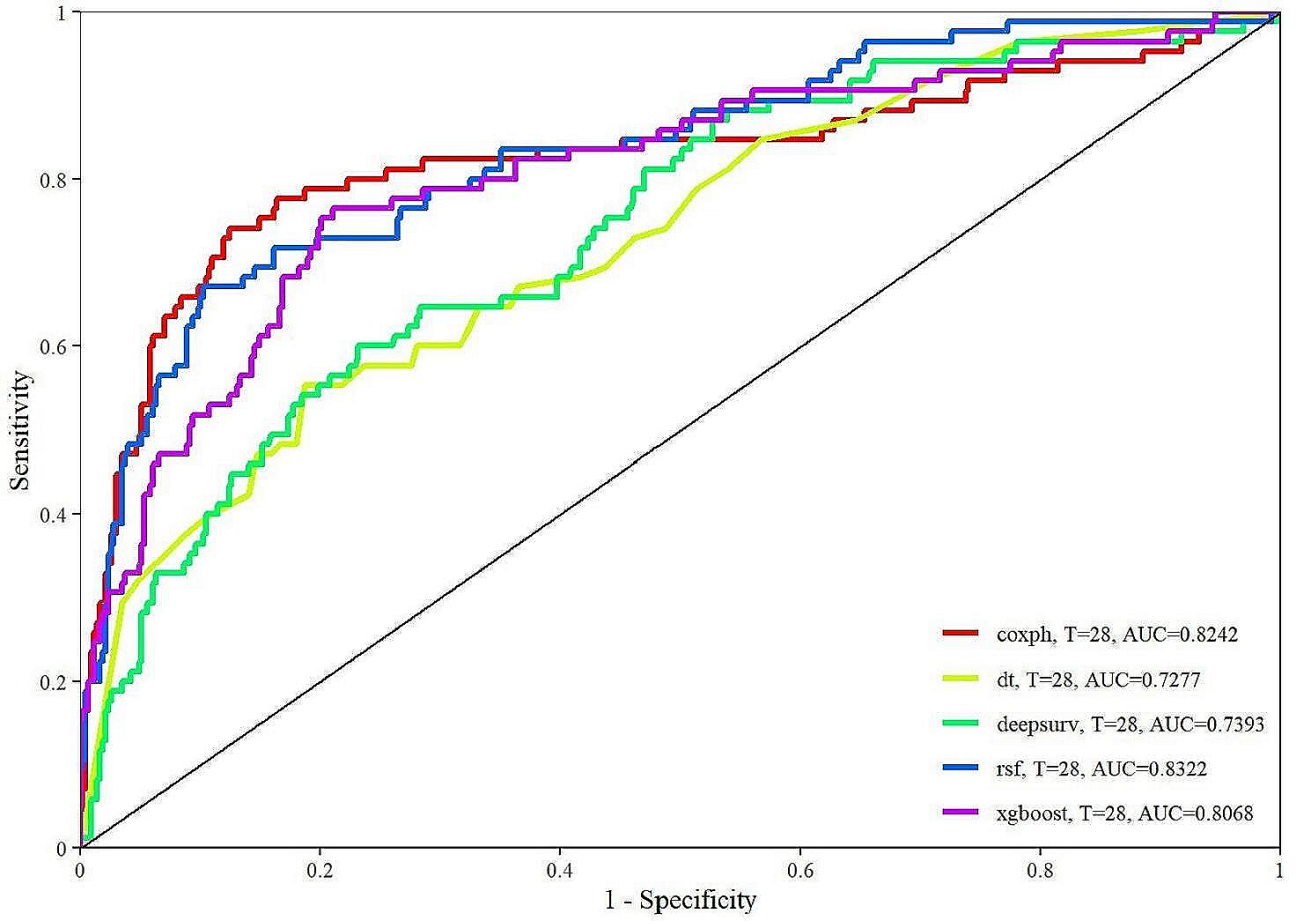
ROC curves of the machine learning algorithms. *coxph* Cox proportional hazards survival learner, *dt* Rpart Survival Trees Survival Learner, *deepsurv* Survival DeepSurv Learner, *rsf* Survival Random Forest SRC Learner, *xgboost* extreme gradient boosting survival learner, *T* days, *AUC* area under the curve

**Fig. 8:**
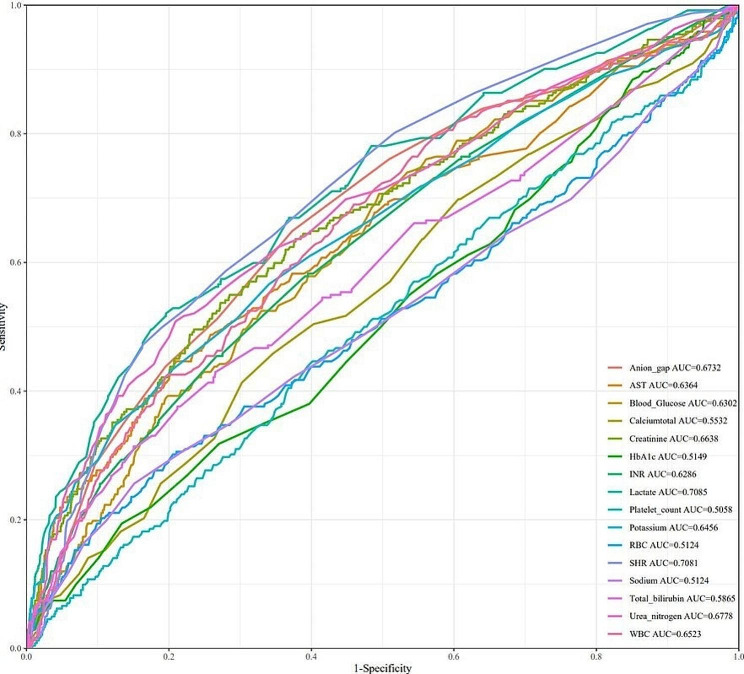
Performance of the SHR and laboratory data. *WBC* white blood cell count, *RBC* red blood cell count, *INR* prothrombin time international normalized ratio, *AST* aspartate aminotransferase, *HbA1c* glycosylated hemoglobin, *SHR* stress hyperglycemia ratio, *AUC* area under the curve

Table S2 shows the hyperparameter, tuning scope and optimal hyperparameter of the four models. Figure 7 displays the ROC curves of various models, and model performance is represented by AUC values. The AUC of coxph was 0.8242, the AUC of dt was 0.7277, the AUC of deepsurv was 0.7393, the AUC of rsf was 0.8322, and the AUC of xgboost was 0.8068. Figure S2 shows the calibration curve of each model. The calibration curves of the coxph, dt, deepsurv, rsf, and xgboost models agree well with the reference line, indicating that they have excellent prediction performance. According to the DCA curve (Figure S3), each model showed a large net benefit, indicating that the established model has robust clinical validity. Figure 8 demonstrates the performance of the SHR and continuous laboratory data. The AUC of the SHR was 0.7081, which was higher than that of blood glucose (AUC = 0.6302) and HbA1c (AUC = 0.5149).

## Discussion

The results of this study indicate a significant association between higher SHR and increased 28-day mortality and in-hospital mortality in critically ill patients with sepsis. These findings remained consistent across age, sex, and subgroup with type 2 diabetes after adjusting for covariates, demonstrating the robustness of the study results. To date, this is the first study of the relationship between the SHR and adverse outcomes in critically ill patients with sepsis.

The Boruta algorithm has become a widely used method in feature selection, determining which features are most important for predicting the target variable by simulating randomness [10]. The feature selection results of the Boruta algorithm in this study suggest that SHR significantly occupies the green area, exhibiting a high Z score in feature selection. This indicates that the SHR may play a crucial role in this study, showing a significant association with the study objectives.

The Boruta algorithm results indicate that the SHR plays an important role in predicting 28-day all-cause mortality in patients with sepsis, but we also recognize that this does not mean that it is a decisive factor. First, the Boruta algorithm is a powerful feature selection method, but it may also be affected by correlations between data features. Therefore, even though the SHR is highly important in the model, this does not necessarily mean that it is the most decisive factor. Second, we found through Cox regression analysis that a greater SHR is associated with an increased risk of 28-day mortality in patients with sepsis, which is consistent with the Boruta algorithm suggesting that the SHR is an important feature. This finding provides evidence that the SHR can be used as a predictor of 28-day all-cause mortality in patients with sepsis. Therefore, we believe that the SHR can be used as a predictor of 28-day all-cause mortality in patients with sepsis.

By incorporating acceptable variables into various machine learning algorithms, the results suggest that these predictive models all demonstrate good performance. It can be reasonably inferred that the stress hyperglycemia ratio may be a significant predictive indicator or influencing factor in this study.

### Relation to previous research

Previous studies have predominantly focused on vascular diseases. Regarding cerebrovascular diseases, research suggests a significant positive correlation between higher SHR quartiles and an increased risk of ischemic stroke transformation to hemorrhage in patients [11]. Additionally, the SHR is independently associated with the severity of post-acute ischemic stroke brain edema, unfavorable functional outcomes, and post-acute ischemic stroke mortality [12]. In cardiovascular diseases, the results of a multicenter prospective study in China demonstrated a correlation between high SHR and increased long-term mortality [13]. In patients without preexisting diabetes, stress hyperglycemia has been shown to be associated with adverse outcomes in stroke [14] and acute myocardial infarction patients [15]. However, stress hyperglycemia does not necessarily represent acute-onset elevated blood glucose; it may reflect poor blood glucose control in patients’ medical history. Therefore, solely exploring the relationship between blood glucose at admission and disease status is not rigorous. Investigating the relationship between the SHR and disease by introducing HbA1c for the correction of past blood glucose levels is a more reasonable approach.

### SHR and sepsis

An elevated SHR indicates a hyperglycemic stress state regardless of past blood glucose levels. Our study confirmed this finding by demonstrating a significant association between a greater SHR and adverse outcomes in critically ill patients with sepsis, irrespective of whether they had type 2 diabetes. This study emphasizes the relationship between the SHR and sepsis, a highlight of our research. First, we explored the potential mechanisms underlying the increased risk of mortality in sepsis patients with high SHRs. Animal experiments suggest that a three-hour infusion of glucose leading to hyperglycemia significantly impairs immune function [16] and activates cytokines while promoting oxidative stress in the liver [17]. High glucose levels can stimulate monocytes to enhance the synthesis and release of interleukin-6 (IL-6) [18], and elevated serum IL-6 exacerbates insulin resistance [19], prompting the liver to release glucose and contributing to hyperglycemia [20]. IL-6 is a pleiotropic cytokine involved in various pathophysiological processes, including inflammation and tissue damage [21], and has been shown to correlate with adverse outcomes in critically ill patients [22]. Additionally, tumor necrosis factor-alpha (TNF-α), a major proinflammatory factor in the pathogenesis of sepsis, is associated with adverse outcomes [23] and can induce insulin resistance [24]. Second, high blood glucose itself may have proinflammatory effects, as lowering blood glucose to 110 mg/dL has been demonstrated to have anti-inflammatory effects in critically ill patients. Our results also show that the risk of adverse events in sepsis patients is minimized when the SHR is approximately 0.85, indicating a potential anti-inflammatory effect of a lower SHR, which plays a role in reducing the occurrence of adverse events.

Furthermore, another critical factor leading to increased mortality in sepsis patients is the occurrence of disseminated intravascular coagulation (DIC). A large, multicenter, prospective study in Japan revealed that the mortality rate of sepsis patients with DIC was twice that of those without DIC [25]. In vitro studies suggest that acute elevation of glucose can cause endothelial damage, promoting abnormal activation of intravascular coagulation, which may lead to sepsis-associated DIC [26]. This could be one of the reasons why a high SHR increases the occurrence of adverse events. Elevated blood glucose promotes the aggregation of monocytes and macrophages [27], leading to the production and release of biologically active molecules such as cytokines, IL-6, and interleukin-8. These factors contribute to inflammation and thrombus formation, thereby promoting DIC.

Additionally, patients with sepsis may develop a hyperglycemic state through multiple mechanisms. First, stress associated with critical illness is characterized by activation of the hypothalamic‒pituitary‒adrenal (HPA) axis and increased release of glucocorticoids, epinephrine, glucagon, and growth hormone [28–31], which can lead to the occurrence of stress hyperglycemia. Second, the release of multiple cytokines, such as interleukin 6 (IL-6) and tumor necrosis factor-α (TNF-α), independently and synergistically with catecholamines promotes hepatic glucose production, whereas interleukin 1 (IL-1) and TNF-α also inhibit insulin release, seemingly in a concentration-dependent manner [32], jointly promoting the occurrence of hyperglycemia. Third, glucose is usually absorbed across cell membranes via carrier-mediated facilitated transport systems [33], and glucocorticoids inhibit glucose transporter 4 (GLUT4), thereby impairing insulin-mediated glucose uptake in skeletal muscle and indirectly promoting the occurrence of hyperglycemia. The above mechanisms indicate that the occurrence of stress hyperglycemia is promoted in patients with sepsis via multiple mechanisms.

The higher SHR and increased risk of all-cause mortality in patients with sepsis may be attributed to the inflammatory response caused by blood glucose fluctuations. First, a higher SHR indicates stress hyperglycemia, which is the result of the complex interaction of hormones such as catecholamines, glucocorticoids, and cytokines [34, 35]. Stress hyperglycemia may lead to increased mitochondrial reactive oxygen species production in endothelial cells, which may cause endothelial dysfunction [36]. Second, stress hyperglycemia contributes to the nonenzymatic glycation of platelet glycoproteins and may be one of the causes of platelet activation, which may lead to an increased risk of thrombosis [37]. The above mechanisms may also contribute to the increased risk of death in patients with sepsis.

### Impact on clinical practice

In a previous study on the association between SHR and critically ill patients with acute myocardial infarction, a higher SHR was only correlated with an increased risk of adverse events in nondiabetic patients, and similar results were not found in diabetic patients [8]. Our study revealed that a greater SHR is associated with an increased risk of 28-day all-cause mortality and in-hospital mortality in critically ill patients with sepsis, regardless of whether they have type 2 diabetes. This suggests that the SHR can be used to predict the risk of adverse events in a broader range of sepsis patients, providing a reliable indicator for clinicians in the diagnosis and treatment of critically ill patients with sepsis.

### Limitations of the study

This study has several limitations. First, this was a retrospective study relying on past records, which may be subject to information bias. Second, not all potential confounding factors could be controlled, limiting causal inferences. Third, sample selection may be influenced by known or unknown factors, resulting in samples that are not representative in certain aspects, potentially affecting the external validity of the study.

## Conclusion

In conclusion, irrespective of the presence of type 2 diabetes, the SHR exhibited a U-shaped relationship with 28-day all-cause mortality and in-hospital mortality in critically ill patients with sepsis. A higher SHR is significantly correlated with an increased risk of adverse events. The SHR can be used to predict adverse outcomes in critically ill patients with sepsis. However, multicenter, prospective studies are still needed to validate these results.

### Supplementary Information

Supplementary Material 1 (JPEG 197 kb)

Supplementary Material 2 (JPEG 86 kb)

Supplementary Material 3 (JPEG 113 kb)

Supplementary Material 4 (DOCX 17 kb)

Supplementary Material 5 (DOCX 17 kb)

## Data Availability

Publicly available datasets were analyzed in this study. These data can be found at https://mimic.mit.edu/.
