# Supplementary figures and images for: Association between the stress hyperglycemia ratio and 28-day all-cause mortality in critically ill patients with sepsis: a retrospective cohort study and predictive model establishment based on machine learning
Source: Cardiovasc Diabetol. 2024 May 9;23:163. doi: 10.1186/s12933-024-02265-4 (PMC11084034; doi:10.1186/s12933-024-02265-4)

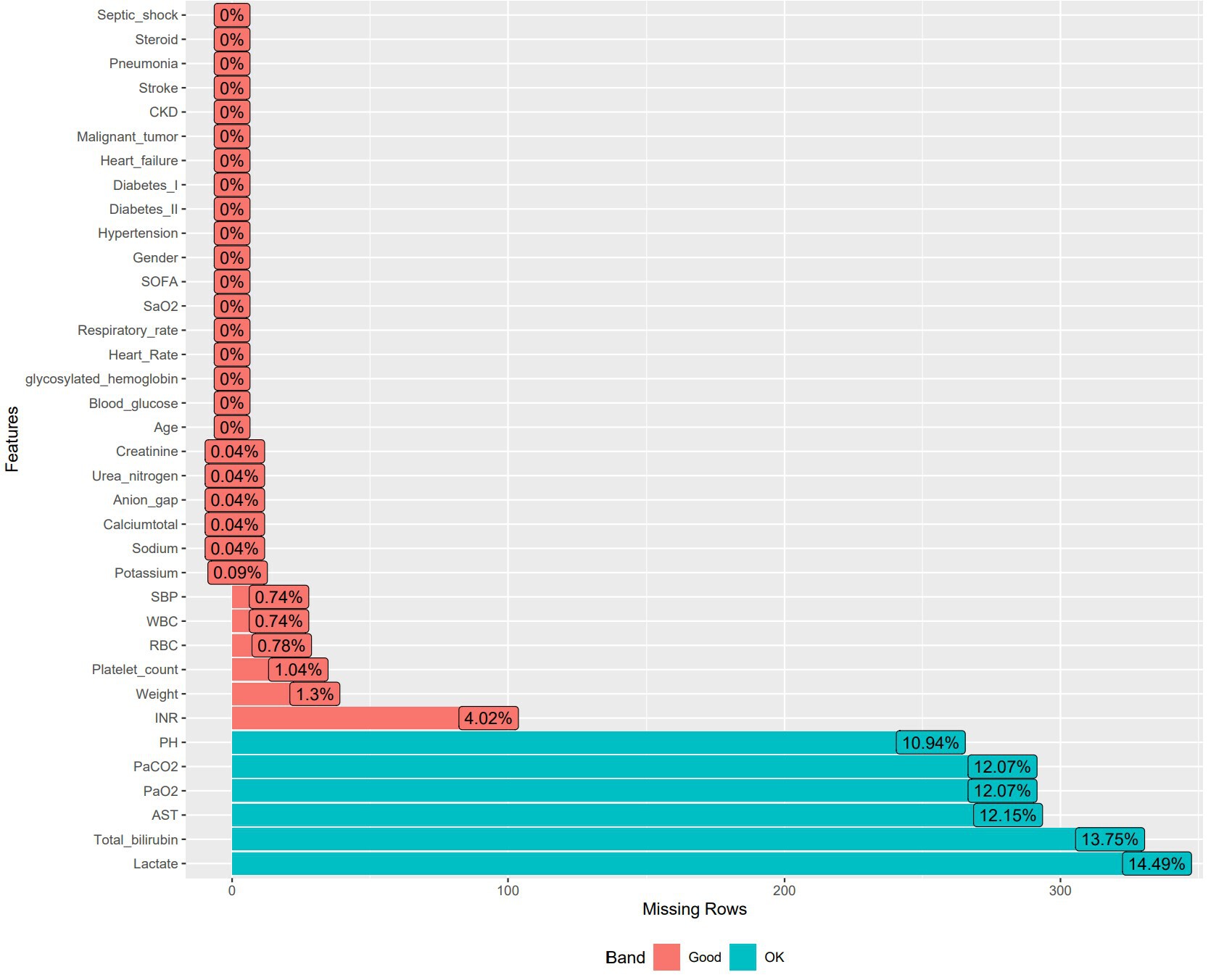

Supplement: Supplementary file 1 — Supplementary Material 1 (JPEG 197 kb) [file 12933_2024_2265_MOESM1_ESM.jpeg]

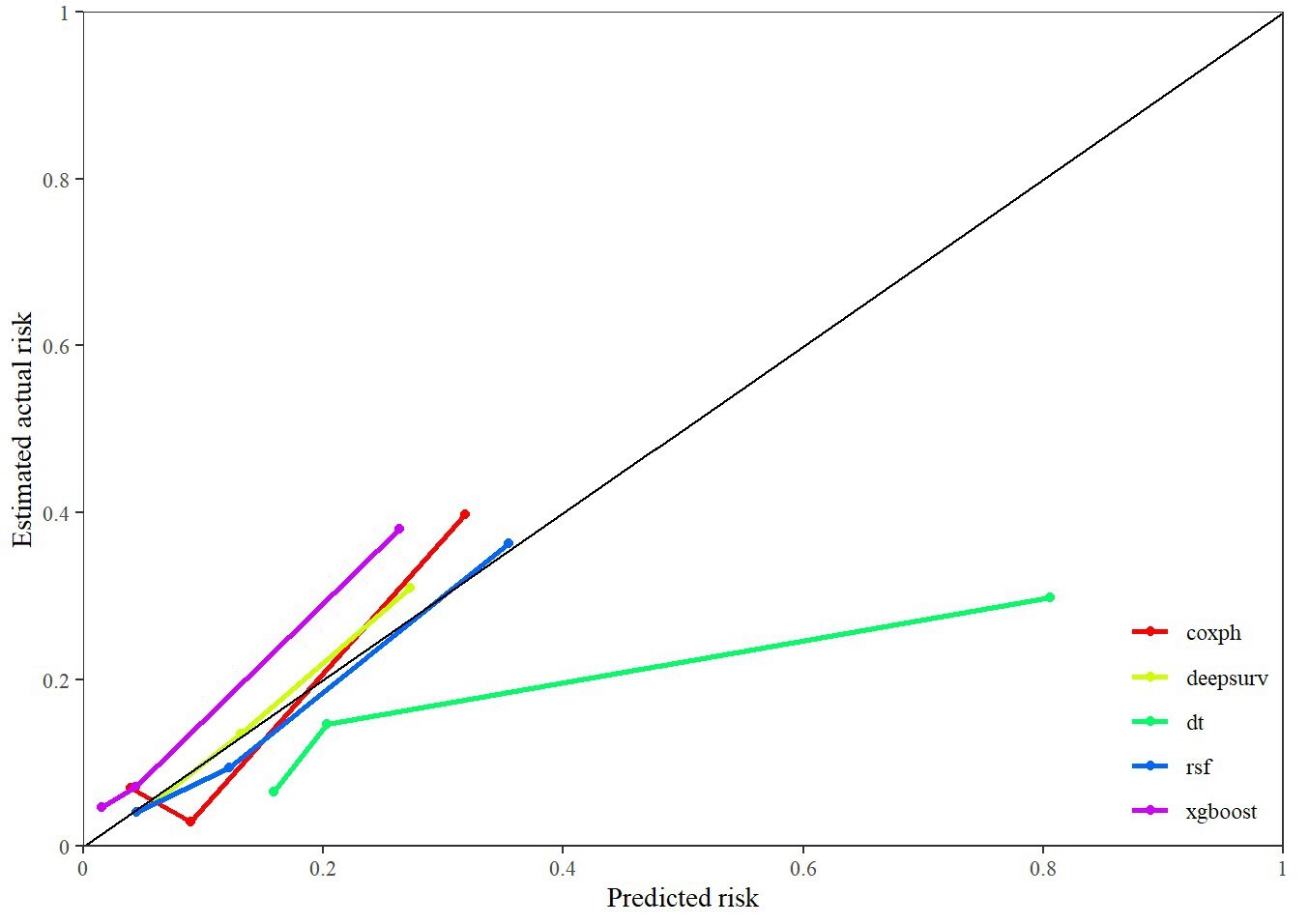

Supplement: Supplementary file 2 — Supplementary Material 2 (JPEG 86 kb) [file 12933_2024_2265_MOESM2_ESM.jpeg]

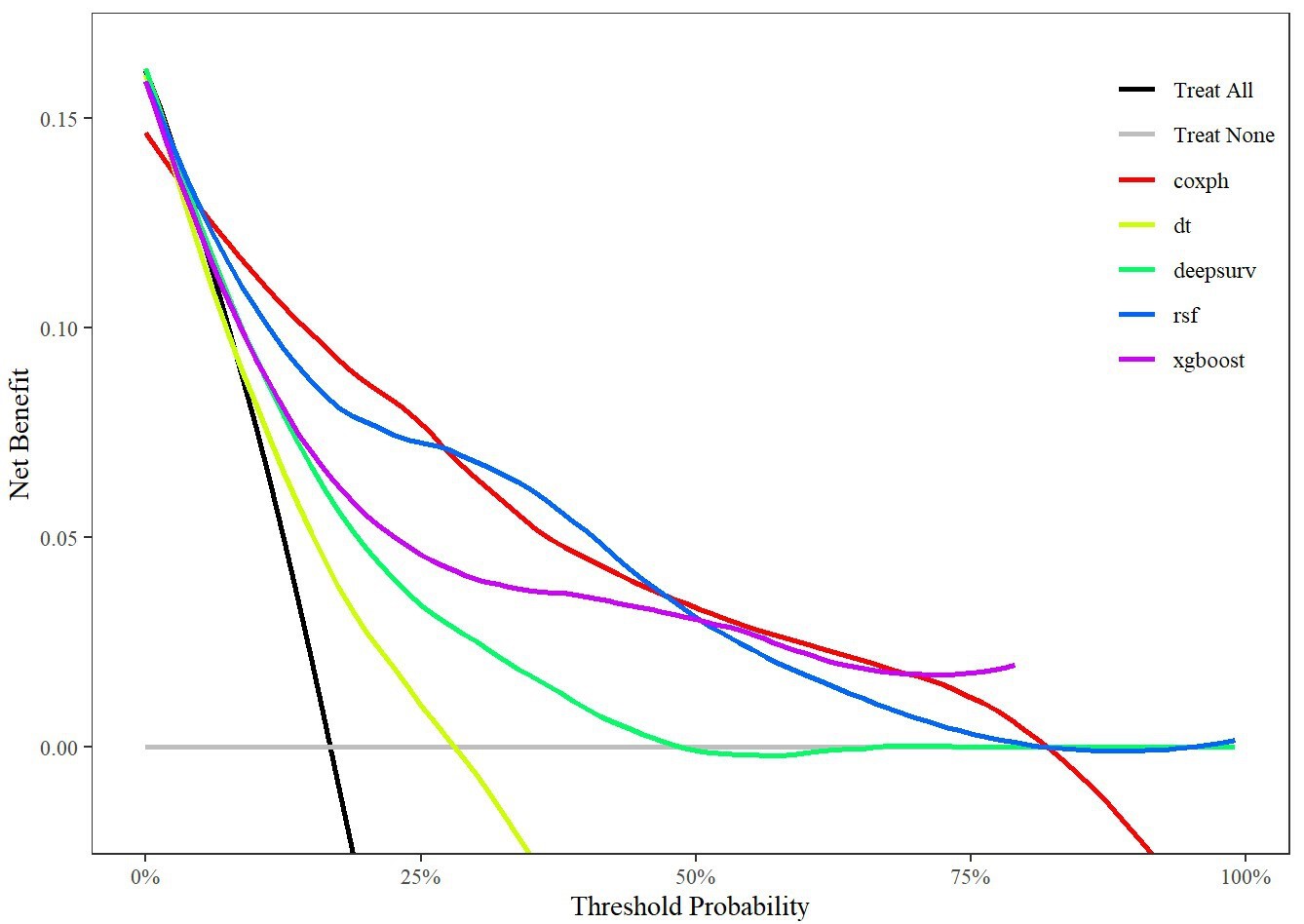

Supplement: Supplementary file 3 — Supplementary Material 3 (JPEG 113 kb) [file 12933_2024_2265_MOESM3_ESM.jpeg]
