# Supplementary material for: Association between the stress hyperglycemia ratio and 28-day all-cause mortality in critically ill patients with sepsis: a retrospective cohort study and predictive model establishment based on machine learning
Source: Cardiovasc Diabetol. 2024 May 9;23:163. doi: 10.1186/s12933-024-02265-4 (PMC11084034; doi:10.1186/s12933-024-02265-4)
Supplement: Supplementary file 4 — Supplementary Material 4 (DOCX 17 kb) [file 12933_2024_2265_MOESM4_ESM.docx]

| **Variables** | **VIF** |
| --- | --- |
| Age | 1.356068 |
| Gender | 1.175649 |
| Weight | 1.277212 |
| WBC | 1.077965 |
| RBC | 1.309909 |
| Platelet count | 1.169391 |
| Sodium | 1.283355 |
| Potassium | 1.366544 |
| Calciumtotal | 1.259391 |
| Anion gap | 2.240794 |
| PH | 2.394336 |
| PaCO2 | 1.79797 |
| PaO2 | 1.154441 |
| Lactate | 2.152979 |
| INR | 1.239303 |
| Total bilirubin | 1.30688 |
| AST | 1.280296 |
| Urea nitrogen | 2.191425 |
| Creatinine | 2.139089 |
| Heart rate | 1.298126 |
| SBP | 1.080716 |
| Respiratory rate | 1.181221 |
| SaO2 | 1.029565 |
| Hypertension | 1.180344 |
| Diabetes II | 1.337552 |
| Diabetes I | 2.987731 |
| Heart failure | 1.248612 |
| Malignant tumor | 1.078074 |
| Sofa | 1.781896 |
| Blood glucose | 1.518239 |
| HbA1c | 1.617488 |
| CKD | 1.397821 |
| Stroke | 1.043551 |
| Pneumonia | 1.082282 |
| Septic shock | 2.139852 |
| Steroid (glucocorticoid) | 1.873953 |

Table S1. Variance inflation factor between variables. Abbreviation: SBP (systolic blood pressure); WBC (white blood cell count); RBC (red blood cell count); PLT (platelet count); PaCO2 (carbon dioxide pressure); PaO2 (arterial oxygen pressure); SaO2 (arterial oxygen saturation); INR (prothrombin time international normalized ratio); AST (aspartate aminotransferase); SOFA (sequential organ failure assessment); HbA1c (glycosylated hemoglobin); CKD (chronic kidney disease); Diabetes I (type 1 diabetes); Diabetes II (type 2 diabetes); SHR (stress hyperglycemia ratio).
