# Supplementary material for: Association between the stress hyperglycemia ratio and 28-day all-cause mortality in critically ill patients with sepsis: a retrospective cohort study and predictive model establishment based on machine learning
Source: Cardiovasc Diabetol. 2024 May 9;23:163. doi: 10.1186/s12933-024-02265-4 (PMC11084034; doi:10.1186/s12933-024-02265-4)
Supplement: Supplementary file 5 — Supplementary Material 5 (DOCX 17 kb) [file 12933_2024_2265_MOESM5_ESM.docx]

For tuning the hyperparameter of machine learning models, a combined approach of grid search and cross-validation is employed. Taking the hyperparameter of dt as an example, we first set out to optimize two hyperparameter: 'cp' and 'minbucket'. For 'cp', the candidate values are set in the range of 0.001 to 0.5 with 10 evenly spaced values, while for 'minbucket', we consider three candidate values: 5, 7, and 9. This results in a total of 30 sets of candidate values. Subsequently, we evaluate the performance of each set of candidate values using cross-validation on the training set to identify the optimal set of hyperparameter. The results of hyperparameter optimization for dt, deepsurv, rsf and xgboost models are presented in Table S2.

| model | hyperparameter | tuning scope | optimal hyperparameter |
| --- | --- | --- | --- |
| dt | cp | (0.001 , 0.5) | 0.001 |
|  | minbucket | (5 , 9) | 9 |
| deepsurv | num | (1 , 3) | 2 |
|  | nodes | (5 , 13) | 5 |
|  | learning_rate | (0 , 0.1) | 0.046 |
|  | dropout | (0 , 0.5) | 0.299 |
|  | weight_decay | (0 , 0.5) | 0.457 |
| rsf | ntree | (200 , 500) | 500 |
|  | mtry | (3 , 5) | 4 |
|  | nodesize | (15 , 21) | 15 |
| xgboost | nrounds | (50 , 100) | 58 |
|  | max_depth | (1 , 2) | 2 |
|  | eta | (0.001 , 1) | 0.244 |

Table S2. Hyperparameter of the four model. dt: Rpart Survival Trees Survival Learner. deepsurv: Survival DeepSurv Learner. rsf: Survival Random Forest SRC Learner. xgboost: Extreme Gradient Boosting Survival Learner.
